# Supplementary material for: The Development of a Series of Genomic DNA Reference Materials with Specific Copy Number Ratios for The Detection of Genetically Modified Maize DBN9936
Source: Foods. 2024 Feb 28;13(5):747. doi: 10.3390/foods13050747 (PMC10930674; doi:10.3390/foods13050747)
Supplement: Supplementary file 1 [file foods-13-00747-s001.zip › foods-2833729-supplementary.pdf]

Development of a series of genomic DNA reference materials with specific copy number ratios for the detection of genetically modified maize DBN9936

Jun Li, Hongfei Gao, Yunjing Li, Shanshan Zhai, Fang Xiao, Gang Wu, * Yuhua Wu *

Key Laboratory of Agricultural Genetically Modified Organisms Traceability of the Ministry of Agriculture and Rural Affairs, Oil Crops Research Institute, Chinese Academy of Agricultural Sciences, Wuhan 430062, China

Table S1. Details of the primers and probes used in this study.

Target	Name	Primer/probe sequence (5'–3')	Amplicon size (bp)
	LF51	CAGGGGCAAGAAAACATC	
DBN9936	LR126	TCTTGTGTGCCCATGAGCCTA	76
	LP79	FAM- TCTTGTGTGCCCATGAGCCTA -BHQ1	
	F	CGGTGGATGCTAAGGCTGATG	
zSSIIb	R	AAAGGGCCAGGTTCATTATCCTC	88
	P	Hex-TAAGGAGCACTCGCCGCCGCATCTG-BHQ1	
	IF	AAGAAAACATCCCAAAACGC	
Insert site	IR	CCATCGCCTCGCCTCGCT	108
	IP	FAM-TGCCGTCGCTTCGGGTCTGTCCCT-TAMRA	

Table S2. Analytical results of the initial homogeneity testing of DBN9936a, DBN9936b, and DBN9936c

Parameter	DBN9936a			DBN9936b			DBN9936c		
	Ratio (%)	DBN9936 (copies/μL)	*zSSIIb* (copies/μL)	Ratio (%)	DBN9936 (copies/μL)	*zSSIIb* (copies/μL)	Ratio (%)	DBN9936 (copies/μL)	*zSSIIb* (copies/μL)
Mean	98.84	34,285	34,727	3.3	1,043.85	31,908.89	1.11	352	31,678
SD	1.26	555	560	0.11	38.94	725.18	0.05	13	616
RSD (%)	1.28	1.62	1.61	3.26	3.73	2.27	4.25	3.73	1.95
F	1.61	1.04	1.86	1.56	1.17	1.35	1.85	1.7	1.11
$F_{(0.05,8,18)}$	2.51	2.51	2.51	2.51	2.51	2.51	2.51	2.51	2.51
Conclusion	homogeneous								

* Corresponding author
E-mail addresses: wugang@caas.cn (G. Wu), wuyuhua@oilcrops.cn (Y-H, Wu).

Table S3. Quantitative data obtained for the Homogeneity assessment.

RM	Sample	DBN9936 (copies/μL)				*zSSIIb* (copies/μL)				DBN9936/*zSSIIb* ratio (%)			
		Rep1	Rep2	Rep3	Mean	Rep1	Rep2	Rep3	Mean	Rep1	Rep2	Rep3	Mean
DBN9936a	1	33840	33640	33920	33800	34620	34180	34180	34327	97.75	98.42	99.24	98.47
	2	34920	34520	34740	34727	34940	34860	35260	35020	99.94	99.02	98.53	99.16
	3	34580	34420	34520	34507	34620	34480	35700	34933	99.88	99.83	96.69	98.80
	4	33980	35100	34040	34373	34700	35200	34380	34760	97.93	99.72	99.01	98.88
	5	34460	34600	34020	34360	35000	35400	34400	34933	98.46	97.74	98.90	98.36
	6	34520	35480	34120	34707	34460	35620	34500	34860	100.17	99.61	98.90	99.56
	7	34620	34120	34660	34467	34840	34400	35420	34887	99.37	99.19	97.85	98.80
	8	34160	34880	34400	34480	35000	35120	34600	34907	97.60	99.32	99.42	98.78
	9	33000	34940	32780	33573	34080	35040	34060	34393	96.83	99.71	96.24	97.60
	10	33780	34360	33800	33980	34900	35640	34840	35127	96.79	96.41	97.01	96.74
	11	34240	34560	34200	34333	34740	34740	34940	34807	98.56	99.48	97.88	98.64
	12	34560	34600	34380	34513	35460	34680	34660	34933	97.46	99.77	99.19	98.81
	13	34460	33500	34420	34127	34500	34840	34820	34720	99.88	96.15	98.85	98.30
	14	33200	34160	34120	33827	34240	34440	34500	34393	96.96	99.19	98.90	98.35
	15	34180	35400	34300	34627	34540	35860	34800	35067	98.96	98.72	98.56	98.75
DBN9936b	1	1130	1104	1064	1099	32040	32040	32060	32047	3.53	3.45	3.32	3.43
	2	1022	1150	1072	1081	32180	32160	32020	32120	3.18	3.58	3.35	3.37
	3	1094	1094	1078	1089	32800	33340	32500	32880	3.34	3.28	3.32	3.31
	4	1122	1034	1104	1087	32160	32080	32120	32120	3.49	3.22	3.44	3.38
	5	1146	1082	1022	1083	32120	32160	32080	32120	3.57	3.36	3.19	3.37
	6	1096	1086	1084	1089	32060	32120	32080	32087	3.42	3.38	3.38	3.39
	7	1038	1064	1038	1047	32160	32080	32100	32113	3.23	3.32	3.23	3.26
	8	1146	1052	1050	1083	32080	30000	32120	31400	3.57	3.51	3.27	3.45
	9	1008	1082	1056	1049	31800	32080	32140	32007	3.17	3.37	3.29	3.28
	10	1010	1052	984	1015	30340	33000	31120	31487	3.33	3.19	3.16	3.23
	11	1048	1018	956	1007	31120	31040	31420	31193	3.37	3.28	3.04	3.23
	12	1032	1024	1076	1044	32100	32020	32140	32087	3.21	3.20	3.35	3.25
	13	1100	1060	1086	1082	32120	32160	32160	32147	3.42	3.30	3.38	3.37
	14	1110	1102	1114	1109	32060	32060	32020	32047	3.46	3.44	3.48	3.46
	15	1056	1096	1014	1055	32020	32080	32120	32073	3.30	3.42	3.16	3.29
DBN9936c	1	302	362	342	335	30000	33200	30600	31267	1.01	1.09	1.12	1.07
	2	362	342	342	349	30780	32000	30600	30793	1.18	1.07	1.12	1.12
	3	366	338	328	344	31920	31580	31720	31740	1.15	1.07	1.03	1.08
	4	316	342	310	323	30640	31080	30420	30713	1.03	1.10	1.02	1.05
	5	374	356	352	361	30940	30800	30080	30607	1.21	1.16	1.17	1.18
	6	356	308	320	328	30460	30100	30000	30187	1.17	1.02	1.07	1.09
	7	330	370	356	352	32800	33080	32800	31967	1.01	1.12	1.09	1.07
	8	338	320	328	329	30680	31160	31720	31187	1.10	1.03	1.03	1.05
	9	358	352	350	353	32040	32040	32120	31393	1.12	1.10	1.09	1.10
	10	340	340	326	335	31720	31940	31720	31793	1.07	1.06	1.03	1.05
	11	326	362	336	341	32000	32040	31000	31680	1.02	1.13	1.08	1.08

12	376	344	346	355	31980	31780	30480	31413	1.18	1.08	1.14	1.13
13	340	334	336	337	31680	31220	31140	31347	1.07	1.07	1.08	1.07
14	350	346	348	348	31220	30000	31360	30860	1.12	1.15	1.11	1.13
15	338	330	330	333	31920	30740	30000	30887	1.06	1.07	1.10	1.08

Table S4. Quantitative data and analytical results for freeze-thaw stability study.

RM	Property value	Freeze-thaw cycle*	Mean	t value	$t_{(0.05, 10)}$	comparison	Conclusion
DBN9936a	DBN9936 (copies/μL)	1	35043			$\|t\| < t_{(0.05, 8)}$	
		2	34543	-1.35	2.23		Stable
		3	34603	-1.05	2.23		Stable
		4	34230	-1.97	2.23		Stable
		5	35467	0.99	2.23		Stable
	zSSIIb (copies/μL)	1	35223		2.23	$\|t\| < t_{(0.05, 8)}$	
		2	34980	-0.74	2.23		Stable
		3	34960	-0.84	2.23		Stable
		4	34487	-1.37	2.23		Stable
		5	35757	1.01	2.23		Stable
	DBN9936/*zSSIIb* (%)	1	99.50			$\|t\| < t_{(0.05, 8)}$	
		2	98.78	0.51	2.23		Stable
		3	98.97	0.53	2.23		Stable
		4	98.35	0.80	2.23		Stable
		5	99.23	0.19	2.23		Stable
DBN9936b	DBN9936 (copies/μL)	1	1050			$\|t\| < t_{(0.05, 8)}$	
		2	1065	-0.43	2.23		Stable
		3	1053	-0.08	2.23		Stable
		4	1085	-0.97	2.23		Stable
		5	999	1.19	2.23		Stable
	zSSIIb (copies/μL)	1	32077			$\|t\| < t_{(0.05, 8)}$	
		2	32760	-0.86	2.23		Stable
		3	31373	0.68	2.23		Stable
		4	32777	-0.89	2.23		Stable
		5	30757	1.22	2.23		Stable
	DBN9936/*zSSIIb* (%)	1	3.27			$\|t\| < t_{(0.05, 8)}$	
		2	3.25	0.33	2.23		Stable
		3	3.36	-1.05	2.23		Stable
		4	3.31	-0.41	2.23		Stable
		5	3.25	0.31	2.23		Stable
DBN9936c	DBN9936 (copies/μL)	1	341			$\|t\| < t_{(0.05, 8)}$	
		2	339	0.11	2.23		Stable
		3	338	0.15	2.23		Stable
		4	348	-0.55	2.23		Stable
		5	348	-0.41	2.23		Stable
	zSSIIb	1	31627				

(copies/μL)	2	31807	-0.39	2.23		Stable
	3	30923	1.48	2.23	$\lvert t \rvert < t_{(0.05,\ 8)}$	Stable
	4	30840	2.15	2.23		Stable
	5	31793	-0.30	2.23		Stable
	1	1.08				
DBN9936/ *zSSIIb* (%)	2	1.06	0.35	2.23		Stable
	3	1.10	-0.39	2.23	$\lvert t \rvert < t_{(0.05,\ 8)}$	Stable
	4	1.13	-1.54	2.23		Stable
	5	1.09	-0.39	2.23		Stable

* DNA solution is stored in a frozen state and used after complete melting at room temperature. A freeze-thaw cycle refers to the process in which the DNA solution undergoes a freezing and thawing process.

Table S5. Data of collaborative characterization obtained from eight laboratories.

RM	Property value	Lab	Rep1	Rep2	Rep3	Rep4	Rep5	Rep6	Rep7	Rep8	mean	SD	RSD（%）
DBN9936a	DBN9936 (copies/μL)	A	33840	33440	32320	34420	33640	33480	34480	34420	33801	755	2.23
		B	33400	33200	33600	33200	33000	32600	32200	32400			
		C	32660	33140	33380	33280	33340	34480	34060	33800			
		D	33560	33380	33200	34780	35020	34820	34760	34040			
		E	33940	33540	34100	33200	35160	34280	34800	34020			
		F	32920	32700	33660	33520	33300	32960	34860	34420			
		G	34480	32940	34360	33260	34300	33900	34940	34640			
		H	33660	34600	34720	34460	34600	35000	33320	33340			
	zSSIIb (copies/μL)	A	35200	34240	34220	35200	34860	33920	34280	34940	34309	846	2.47
		B	33600	34000	33800	34400	33600	32600	32400	32800			
		C	33500	33280	33860	33160	34300	33940	33900	34100			
		D	34800	33740	33780	34860	35660	35480	35200	33700			
		E	34920	34180	34700	33860	35560	34320	35700	35260			
		F	32480	33640	32940	34360	34000	33700	33600	35660			
		G	34560	33220	34420	34040	35260	34520	35720	35240			
		H	34760	35020	34980	34520	35500	35320	34960	33560			
	DBN9936/*zSSIIb* (%)	A	96.14	97.66	94.45	97.78	96.5	98.7	100.58	98.51	98.42	1.35	1.37
		B	99.4	97.65	99.41	96.51	98.21	100	99.38	98.78			
		C	97.49	99.58	98.58	100.36	97.2	101.59	100.47	99.12			
		D	96.44	98.93	98.28	99.77	98.21	98.14	98.75	101.01			
		E	97.19	98.13	98.27	98.05	98.88	99.88	97.48	96.48			
		F	99.57	97.86	99.27	97.96	98.59	98.81	98.1	96.52			
		G	99.77	99.16	99.83	97.71	97.28	98.2	97.82	98.3			
		H	96.84	98.8	99.26	99.83	97.46	99.09	95.31	99.34			
DBN9936b	DBN9936 (copies/μL)	A	1080	1072	984	1032	1072	1054	1056	1032	1050	34	3.24
		B	1048	1040	986	1016	974	1004	1054	1096			
		C	1074	1044	1058	1044	1002	1036	1070	1040			

		D	1092	1046	1052	1058	1088	1052	1080	1116			
		E	1046	1056	1088	1070	1096	1102	1076	1066			
		F	1026	1004	1040	1022	1032	1006	1038	1044			
		G	1012	1064	1060	1028	1020	1018	994	1104			
		H	1064	1020	1026	1064	1100	1046	1078	1138			
	zSSIIb (copies/μL)	A	30180	29920	28740	30800	29860	31540	29060	29220	31006	1142	3.68
		B	31200	30800	30600	29200	29800	29200	29200	31320			
		C	31120	31280	30920	30360	31140	31300	30760	31460			
		D	31820	32200	31560	31960	32160	31920	32540	32560			
		E	32000	31740	31760	31400	31380	32900	32160	32180			
		F	28360	30300	30540	29580	30320	29280	29440	28900			
		G	30760	30840	31580	32060	31220	31360	30700	32060			
		H	32860	30500	32500	31380	31420	32360	32200	32640			
	DBN9936/z*SSIIb* (%)	A	3.58	3.58	3.42	3.35	3.59	3.34	3.63	3.53	3.39	0.11	3.24
		B	3.36	3.38	3.22	3.48	3.27	3.44	3.61	3.5			
		C	3.45	3.34	3.42	3.44	3.22	3.31	3.48	3.31			
		D	3.43	3.25	3.33	3.31	3.38	3.3	3.32	3.43			
		E	3.27	3.33	3.43	3.41	3.49	3.35	3.35	3.31			
		F	3.62	3.31	3.41	3.46	3.4	3.44	3.53	3.61			
		G	3.29	3.45	3.36	3.21	3.27	3.25	3.24	3.44			
		H	3.24	3.34	3.16	3.39	3.5	3.23	3.35	3.49			
DBN9936c	DBN9936 (copies/μL)	A	338	390	360	356	348	374	352	370	351	15	4.27
		B	348	328	328	344	342	340	328	340			
		C	360	344	334	354	356	342	350	346			
		D	342	342	346	348	336	324	356	348			
		E	348	364	344	338	352	352	382	348			
		F	336	318	340	360	374	348	360	338			
		G	362	368	370	354	366	348	366	346			
		H	344	378	356	338	380	378	354	350			
	zSSIIb (copies/μL)	A	30400	31600	31080	31720	30100	31060	31740	31720	31059	831	2.68
		B	29600	29400	29600	31000	30400	29600	28600	30000			
		C	32180	31060	31160	31020	31600	31580	31740	31180			
		D	30100	30180	29880	31180	29820	30520	30260	30880			
		E	30480	31840	32660	32540	31720	31200	31920	30860			
		F	30820	29940	30520	31060	31320	30840	31300	30680			
		G	32140	31460	31800	31780	31180	31260	30860	31340			
		H	32160	31700	31760	31720	31720	31600	31720	31920			
	DBN9936/z*SSIIb* (%)	A	1.11	1.21	1.14	1.15	1.19	1.18	1.17	1.17	1.13	0.04	3.54
		B	1.18	1.12	1.11	1.11	1.13	1.15	1.15	1.13			
		C	1.12	1.07	1.08	1.14	1.15	1.08	1.1	1.11			
		D	1.14	1.13	1.16	1.24	1.13	1.06	1.18	1.13			
		E	1.14	1.14	1.05	1.04	1.11	1.13	1.2	1.13			
		F	1.09	1.06	1.11	1.16	1.19	1.13	1.15	1.1			

		G	1.13	1.17	1.16	1.1	1.15	1.12	1.16	1.1			
		H	1.07	1.19	1.12	1.07	1.2	1.2	1.12	1.1			
